# Identification of neurological complications in childhood influenza: a random forest model

**DOI:** 10.1186/s12887-024-04773-4

**Published:** 2024-05-20

**Authors:** Suyun Li, Weiqiang Xiao, Huixian Li, Dandan Hu, Kuanrong Li, Qinglian Chen, Guangming Liu, Haomei Yang, Yongling Song, Qiuyan Peng, Qiang Wang, Shuyao Ning, Yumei Xiong, Wencheng Ma, Jun Shen, Kelu Zheng, Yan Hong, Sida Yang, Peiqing Li

**Affiliations:** 1 Pediatric Emergency Department, Guangzhou Women and Children’s Medical Center, Guangzhou Medical University, Guangzhou, 510623 China; 2 Department of Radiology, Guangzhou Women and Children’s Medical Center, Guangzhou Medical University, Guangzhou, 510623 China; 3 Institute of Pediatrics, Guangzhou Women and Children’s Medical Center, Guangzhou Medical University, Guangzhou, 510623 China; 4 Pediatric Neurology Department, Guangzhou Women and Children’s Medical Center, Guangzhou Medical University, Guangzhou, 510623 China; 5 Neuroelectrophysiology Department, Guangzhou Women and Children’s Medical Center, Guangzhou Medical University, No.9 Jinsui Road, Guangzhou, 510623 China

**Keywords:** Influenza, Children, Complications, Encephalitis, Acute febrile, Acute necrotizing encephalopathy, Predictive model

## Abstract

**Background:**

Among the neurological complications of influenza in children, the most severe is acute necrotizing encephalopathy (ANE), with a high mortality rate and neurological sequelae. ANE is characterized by rapid progression to death within 1–2 days from onset. However, the knowledge about the early diagnosis of ANE is limited, which is often misdiagnosed as simple seizures/convulsions or mild acute influenza-associated encephalopathy (IAE).

**Objective:**

To develop and validate an early prediction model to discriminate the ANE from two common neurological complications, seizures/convulsions and mild IAE in children with influenza.

**Methods:**

This retrospective case-control study included patients with ANE (median age 3.8 (2.3,5.4) years), seizures/convulsions alone (median age 2.6 (1.7,4.3) years), or mild IAE (median age 2.8 (1.5,6.1) years) at a tertiary pediatric medical center in China between November 2012 to January 2020. The random forest algorithm was used to screen the characteristics and construct a prediction model.

**Results:**

Of the 433 patients, 278 (64.2%) had seizures/convulsions alone, 106 (24.5%) had mild IAE, and 49 (11.3%) had ANE. The discrimination performance of the model was satisfactory, with an accuracy above 0.80 from both model development (84.2%) and internal validation (88.2%). Seizures/convulsions were less likely to be wrongly classified (3.7%, 2/54), but mild IAE (22.7%, 5/22) was prone to be misdiagnosed as seizures/convulsions, and a small proportion (4.5%, 1/22) of them was prone to be misdiagnosed as ANE. Of the children with ANE, 22.2% (2/9) were misdiagnosed as mild IAE, and none were misdiagnosed as seizures/convulsions.

**Conclusion:**

This model can distinguish the ANE from seizures/convulsions with high accuracy and from mild IAE close to 80% accuracy, providing valuable information for the early management of children with influenza.

**Supplementary Information:**

The online version contains supplementary material available at 10.1186/s12887-024-04773-4.

## Introduction

 Neurological complications of influenza are uncommon, but permanent sequel and death are not rare [1]. Seizures or febrile convulsions and influenza-associated encephalopathy (IAE) are two of the most frequently reported neurological complications and the common cause of hospital admissions among children with influenza. During the hospitalization, some children may have seizures/convulsions alone or mild IAE, but some children may quickly develop into the most severe category of IAE, acute necrotizing encephalopathy (ANE), with a high frequency of neurologic sequelae (33–50%) and mortality rate (around 30%) [2–4]. The majority of the patients with IAE falls in the age of 1 to 5-year-old [5], while ANE typically occurs in children < 5 years of age and is characterized by rapid progression to encephalopathy, coma, or death within 1–2 days from onset [6–8]. Therefore, early diagnosis and intervention for ANE are crucial.

ANE is defined as acute fever, frequent convulsions, acute disturbance of consciousness even coma, and multiple organ failure, with a risk of death [6–8]; biochemistry changes are not specific [4], but imaging shows brain edema and necrosis of thalamus and other deep brain structures [4, 9, 10]. Generally, contrast-enhanced computed tomography (CT) can detect ring-shaped enhancement of the thalamus and deep brain white matter 3 days after illness onset [11]. However, irregular high-density shadows in the hypothalamic mottled low-density area appeared until 7 days after illness onset, while no abnormal lesions were found in patients who died within 30 h. Similarly, significant gray matter damage volume changes can be observed using conventional magnetic resonance imaging (MRI) [12]. Three days after onset, the thalamus display a concentric-ring pattern in the T1-weighted image (T1WI). In the second week, the T1WI reveals ring-shaped increased signal intensity in the thalamus. Moreover, diffusion-weighted imaging (DWI) and apparent diffusion coefficient (ADC) map show a concentric pattern in the acute phase of typical cases [13]. These imaging studies showed that there might be no abnormalities found on brain CT or MRI in the early stage of ANE, which is one of the reasons for the low early diagnosis rate of ANE. When the typical ANE brain imaging is found, the patient may miss the opportunity for early intervention and progress to death or severe sequelae. Therefore, finding clinical indicators for early identification of ANE from patients with influenza neurological complications is necessary. However, the knowledge about the reliable and early diagnosis of ANE is limited. The ANE can be controlled, and the prognosis can be improved if early treatment is undertaken, as Low brain temperature, antiviral medication, immunoglobulin, glucocorticoids, and plasma exchange [4]. Before rapid antigen tests for influenza widely available, antiviral treatment was usually given until the positive results of nucleic acid PCR return (probably 24 h after sampling in our medical center). Many ANE patients rapidly deteriorated into coma or other consciousness disorders before positive results or antiviral treatment taken. Therefore, Indicators that can be quickly obtained in the emergency department, such as detailed demographic, clinical characteristics, biochemistry and hematologic indicators in serum samples, may have clinical significance for early diagnosis of ANE. Compared with seizures/convulsions alone or mild IAE, ANE is a relatively rare entity that can masquerade from febrile convulsions or other neurological complications in the early phase [14]. Most recent studies related to the clinical characteristics of ANE were limited by small sample sizes or based on case reports/series, and only recruited ANE patients [3, 15–18]. To date, there is a lack of studies for discriminating the ANE from two common neurological complications, seizures/convulsions and IAE, in children with influenza. Random forest (RF) is a robust and commonly-used machine learning algorithm that can be used for disease diagnosis and classification, and is good at describing the relationship between independent and dependent variables with high flexibility and sufficient accuracy [19]. Therefore, the objective of our study was to develop and validate an early prediction model using a random forest model to distinguish ANE from seizures/convulsions alone and mild IAE in children with influenza.

## Materials and methods

### Study design and patients

To develop the multivariable diagnostic model, we designed and implemented a retrospective case-control study. This study was approved by the Ethics Committee of Guangzhou Women and Children Medical Center ([2019]38,201). All patients signed an informed consent form upon admission. Reporting of this study has followed the Transparent Reporting of a Multivariable Prediction Model for Individual Prognosis or Diagnosis (TRIPOD) statement.

In Guangzhou Women and Children’s Medical Center in Guangzhou (GWCMC) that provided tertiary care, patients hospitalized with influenza virus infection and had neurological manifestations at hospitalization between November 2012 to January 2020 were enrolled. The inclusion criteria were: (1) age < 18 years; (2) laboratory confirmation of influenza; and (3) neurological manifestations, such as convulsion, acute cognitive impairment, acute disturbance of consciousness, coma, and abnormalities in the cerebrospinal fluid examination or head imaging. Patients were considered as ineligible if they met the following exclusion criteria: (1) admission > 7 days after onset; (2) co-infected with other pathogens; (3) comorbidities like brain trauma, sequelae of viral encephalitis, or metabolic diseases; (4) missing data > 30%; or (5) neurological complications other than ANE, seizures/convulsions, and mild IAE.

### Outcome to be predicted and reference standard

There were three outcomes for all subjects met the inclusion criteria: seizure, mild IAE and ANE. Seizures/convulsions related to influenza was defined as convulsive seizures during fever, consciousness after the convulsion, a maximum of two seizures/convulsions events, and no abnormalities in the cerebrospinal fluid (CSF) examination and brain imaging, if done. The type of seizures/convulsions is a generalized tonic-clonic seizure, including very few patients with previously diagnosed febrile seizures presenting as atonic and binocular staring. As the pathogenesis of IAE and ANE is not fully understood, there is an overlap between the symptoms of the two disorders [20–22]. In order to avoid confusion, we defined the IAE patients matching the following criteria as mild IAE enrolled in the study, that presented short term convulsions(< 5 min), coma lasting more than 24 h [6, 23], but recovered completely without abnormal results in CSF and neuroimaging. ANE was defined as acute fever, frequent convulsions(≥ 3 times), coma, multiple organ failure, even death [2, 7, 8]; imaging shows brain edema and necrosis of the thalamus and other deep brain structures [4, 9, 10].

### Candidate predictors

Detailed demographic, clinical characteristics at admission, and biochemistry and hematologic indicators in serum sample of the patients were extracted from the structured electronic medical records system (EMRS) (Table 1). In addition, the first blood sample for measurement of hematologic indicators was obtained from patients immediately after admission.

**Table 1:** Demographic, clinical, and laboratory data of the patients

Variable, median (IQR) or as shown	Reference Range		Seizure, *n* = 278	Mild IAE, *n* = 106	ANE, *n* = 49	*P*
Sex (male), n (%)			197 (70.9)	73 (68.9)	24 (49.0)	0.010^*^
Age (years)			2.6 (1.7,4.3)	2.8 (1.5,6.1)	3.8 (2.3,5.4)	0.018^*^
Onset (days)			2 (1,3)	3 (2,4)	3 (2,5)	< 0.001^*^
Fever (days)			2 (1,3)	2 (1,4)	3 (2,4)	< 0.001^*^
Convulsions, n (%)						
None			4^a^ (1.4)	30 (28.3)	17 (34.7)	< 0.001^*^
1 to 2, times			259 (93.2)	35 (33.0)	16 (32.6)	
≥ 3, times			15 (5.4)	41 (38.7)	16 (32.6)	
Unconsciousness(coma), n (%)			17^b^ (6.1)	27 (25.5)	31 (63.3)	< 0.001^*^
Influenza virus type, n (%)						
Flu A			168 (60.4)	68 (64.1)	18 (36.7)	0.009^*^
H1N1			22 (7.9)	11 (10.4)	9 (18.4)	
Flu B			88 (31.7)	27 (25.5)	22 (44.9)	
Cholinesterase (U/L)	4650–12,220		8165.8 (7644,8451)	7994 (7096,8555)	6882.5 (5821,8027)	< 0.001^*^
ALT (U/L)	7–30		17 (13,24)	20 (14,29)	65 (21,456)	< 0.001^*^
γ-GT (U/L)	5–19		11 (9,13)	12 (11,16.3)	18 (12,31)	< 0.001^*^
ALP (U/L)	143–406		222.7 (189,236)	196 (148,243)	130 (93,174)	< 0.001^*^
Amylase (U/L)	35–135		63.1 (48,63.1)	64.5 (43,79)	75 (48,153.4)	0.002^*^
AST (U/L)	14–44		41 (33,52.3)	44 (34,67)	117 (55,615)	< 0.001^*^
Globulin (g/L)	15–34		23.6 (21.6,25.7)	25 (22,26.6)	26 (21.5,39.4)	0.001^*^
CK-CKMB (U/L)	40–390		203 (106,354.5)	150.5 (85,408)	336 (97,688)	0.095
Lactic dehydrogenase (U/L)	159–322		291 (260,326)	301 (265,343)	508 (356,1283)	< 0.001^*^
A/G	1.2-3.0		1.9 (1.7,2)	1.7 (1.5,2)	1.4 (1.1,1.8)	< 0.001^*^
α-HBDH (U/L)	206–309		253 (224,276)	257.5 (209,276)	398 (282,731)	< 0.001^*^
CK (U/L)	45–390		160 (110,298)	171 (107,395)	401 (132,968)	0.004^*^
Calcium (mmol/L)	1.1–1.5		2.3 (2.2,2.4)	2.3 (2.2,2.4)	2.2 (2.1,2.3)	< 0.001^*^
CKMB (U/L)	0–5		23 (18,30)	26.5 (19,36.4)	61 (27,81)	< 0.001^*^
Total protein (g/L)	61–79		66.5 (63.5,69.6)	66.8 (63.7,70.9)	66.8 (57,75.4)	0.900
Albumin (g/L)	39–54		42.6 (40.8,44.7)	42.1 (40.4,44.3)	38.1 (33.5,41.7)	< 0.001^*^
Total bile acid (µmol/L)	0.5–10.0		7.2 (4.6,10.7)	6.5 (3.8,10.7)	5.4 (2.8,15)	0.352
hs-CRP, (mg/L)	0–6		11.1 (3.2,11.1)	7.8 (1.6,16.3)	10.2 (3.2,29.2)	0.601
Uric acid (µmol/L)	90–420		291.5 (235,332)	285 (235,309)	319 (178,454)	0.262
IBIL (µmol/L)	2-13.7		3.1 (2.1,4.2)	2.8 (2,3.8)	3.5 (1.8,4.8)	0.389
DBIL (µmol/L)	0–7		1.5 (1.1,1.9)	1.6 (1.2,2.2)	2 (1.2,3.7)	0.002^*^
Lipase (U/L)	13–60		35.2 (32,38)	35 (28,38)	41.9 (26,65)	0.074
Creatinine (µmol/L)	19–44		26.5 (23,31)	28 (22,31)	33 (24,43)	0.002^*^
TBIL (µmol/L)	2–17		4.7 (3.5,6.2)	4.5 (3.4,5.6)	5.5 (3.5,9)	0.225
Thrombin time (s)	14–21		17 (16.3,17.8)	17.5 (16.5,18)	19.1 (17.1,20.2)	< 0.001^*^
Prothrombin time (s)	11–15		14 (13.2,14.8)	14.5 (13.4,15.2)	16.2 (14,17.4)	< 0.001^*^
Fibrinogen (g/L)	2–4		2.8 (2.4,3.1)	2.9 (2.5,3.1)	2.9 (2.2,3.3)	0.692
Coagulation activity (%)	70–120		86.2 (80,88)	85.4 (76,95)	72 (62,88)	< 0.001^*^
INR	0.8–1.5		1.1 (1,1.2)	1.1 (1,1.2)	1.3 (1.1,1.5)	< 0.001^*^
CRP (mg/L)	0–8		4.6 (1.3,7.1)	5.7 (1,14.8)	14.5 (2.4,21.4)	< 0.001^*^
RBC (10^12^/L)	4.1–5.5		4.5 (4.2,4.7)	4.4 (4.1,4.7)	4.2 (3.7,4.5)	< 0.001^*^
Monocytes (10^9^/L)	0.12–0.93		0.6 (0.4,0.8)	0.5 (0.4,0.8)	0.5 (0.2,0.8)	0.152
Monocytes% (%)	2–11		8 (5.9,10.3)	8 (6,10.9)	6 (3,8.3)	0.002^*^
Lymphocytes (10^9^/L)	1.8–6.3		2 (1.1,3.1)	1.9 (1.2,3.3)	1.6 (0.7,2.4)	0.116
Lymphocytes% (%)	26–67		29 (15.5,49.2)	29 (14.9,46)	21 (11,31)	0.020^*^
Large platelet ratio (%)	13–43		22.2 (17.8,27.2)	23.9 (17.9,29.2)	25.1 (19.5,29.8)	0.043^*^
MCV (fL)	76–88		80.4 (77.4,83.3)	81 (77.5,83.9)	82.2 (78.2,85.6)	0.043^*^
Eosinophils (10^9^/L)	0.04–0.74		0 (0,0)	0 (0,0)	0 (0,0)	0.001^*^
MPV (fL)	7.6–13.2		9.7 (9.2,10.4)	9.9 (9.2,10.6)	10 (9.4,10.7)	0.088
RBC distribution width (%)	11.5–14.5		38 (36.4,40.1)	37.9 (36.7,39.5)	40.3 (38.3,42.7)	< 0.001^*^
Thrombocytocrit (%)	0.1–0.5		0.2 (0.2,0.3)	0.2 (0.2,0.3)	0.2 (0.2,0.3)	0.741
Mean hemoglobin concentration (pg)	309–359		332 (324,342)	333 (326,342)	332.5 (324,341)	0.642
Neutrophils (10^9^/L)	1.3–6.7		4.2 (2.1,6.7)	3.9 (2,6.5)	5.8 (3.4,7.7)	0.058
Neutrophils% (%)	23–64		59 (38,75)	57.1 (37,75)	70 (55,81)	0.010^*^
Blood platelets (10^9^/L)	187–475		230 (186,279)	233.5 (173,293)	169 (102,275)	0.022^*^
PDW	14.8–17.2		10.3 (9.5,11.6)	10.8 (9.5,11.9)	11.1 (10,12.3)	0.013^*^
RBC distribution width, CV (%)	11.5–14.5		13.2 (12.6,14)	13.1 (12.4,13.7)	13.7 (12.9,14.4)	0.007^*^
WBC (10^9^/L)	4.9–12.7		7.2 (5.3,9.8)	7.1 (5.3,9.7)	8.1 (5.3,10)	0.779
Oxygen partial pressure (kPa)	> 10.64		9.2 (5.9,20.3)	8.8 (5.9,12)	15.3 (11.3,20.3)	< 0.001^*^
Urea (mmol/L)	2.1–7.1		3.7 (3.3,4)	3.7 (3,3.7)	5.9 (4,7.9)	< 0.001^*^
Procalcitonin (ng/ml)	< 0.1		1.2 (0.3,2.4)	3.2 (0.6,4)	13.8 (0.5,13.8)	< 0.001^*^
Potassium (mmol/L)	3.4–5.7		3.9 (3.6,4.2)	3.8 (3.5,4.1)	3.8 (3.4,4.1)	0.216
BEECF (mmol/L)	-2-+3		-2.7 (-3.7,-1.8)	-2.9 (-4.2,-1.1)	-5 (-9.3,-1.3)	0.019^*^
Carbon dioxide (mmol/L)	18.5–24.5		22.8 (20.7,24.8)	22.5 (20.4,24.2)	21.7 (16.5,24.8)	0.040^*^
Hematocrit (%)	34–45		36.3 (34.5,38.8)	37 (35,39.4)	34 (31,38)	0.003^*^
Standard ionic calcium (mmol/L)	1.1–1.5		1.2 (1.2,1.2)	1.2 (1.1,1.2)	1.2 (1.1,1.2)	0.460
Lactic acid (mmol/L)	0.9–1.7		1.8 (1.2,2.4)	1.7 (1.2,2.2)	1.7 (1.1,2.2)	0.941
Standard bicarbonate (mmol/L)	21.3–24.8		22.9 (21.9,23.8)	22.7 (21.7,24.2)	21.4 (18.5,24.1)	0.029^*^
Whole blood residual base (mmol/L)	-3-+3		-2.1 (-3.5,-0.7)	-2.2 (-3.3,-0.7)	-4.3 (-7.7,-0.8)	0.013^*^
Calcium ions (mmol/L)	1.1–1.5		1.2 (1.1,1.2)	1.2 (1.1,1.2)	1.2 (1.1,1.2)	0.315
Acidity (pH)	7.35–7.45		7.4 (7.4,7.4)	7.4 (7.4,7.4)	7.4 (7.3,7.4)	0.107
PCO2 (kPa)	4.66–5.99		5 (4.5,5.3)	5 (4.5,5.5)	4.3 (3.9,5.2)	0.008^*^
Glucose (mmol/L)	4.1–5.9		5.9 (5.1,6.9)	6.1 (5.2,6.8)	5.8 (5,7.4)	0.871
Sodium (mmol/L)	138–144		135.6 (133,138)	135 (132.4,137)	137.7 (134,141)	0.001^*^
Hemoglobin (g/L)	115–150	118 (112,124)		119.5 (112,128)	116 (102,129)	0.184
Immunoglobulin A (g/L)	0.63–1.79	0.7 (0.4,1)		0.8 (0.4,1.1)	0.8 (0.4,1)	0.059
Immunoglobulin E (IU/ML)	0-200		92.6 (23,128)	92 (27,177.1)	104 (23.9,215.5)	0.180
Immunoglobulin G (g/L)	6.36–14.04		8 (6.6,9.2)	9.1 (7.3,10.4)	11.5 (7.6,12.7)	< 0.001^*^
Immunoglobulin M (g/L)	0.29–1.41		1.1 (0.8,1.3)	1.1 (0.7,1.3)	1.2 (0.9,1.3)	0.260
Complement C3 (g/L)	0.8–1.5		0.9 (0.8,0.9)	0.9 (0.8,1)	0.8 (0.6,0.9)	< 0.001^*^
Complement C4 (g/L)	0.13–0.43		0.2 (0.2,0.2)	0.2 (0.2,0.3)	0.2 (0.2,0.3)	0.011^*^

*IQR *Interquartile range, *ALT *alanine aminotransferase, *γ-GT *γ-glutamyltransferase, *ALP *alkaline phosphatase, *AST *aspartate aminotransferase, *A/G *albumin/globulin ratio, *CK *creatinine kinase, *CKMB *Creatinine kinase MB, *α-HBDH *α-hydroxybutyric dehydrogenase, *hs-CRP *High-sensitivity C-reactive protein, *IBIL *Indirect bilirubin, *TBIL *Total bilirubin, *INR *International normalized ratio, *CRP *C-reactive protein, *RBC *Red blood cells, *MCV *Mean corpuscular volume, *SD *Standard deviation, *PDW *Platelet distribution width, *CV *Coefficient of variation, *WBC *White blood cells, *BEECF *Extracellular residual base, *PCO2 *Carbon dioxide pressure

*P <0.05

^a^Four patients with a history of febrile seizures presented with transient atonic and binocular staring rather than generalized tonic clonic

^b^There were 17 cases presented in complex febrile seizures and febrile status epilepticus (FSE), whose consciousness without fully recovering between seizures [24].

### Prediction model development

The prediction model was developed based on an RF classification algorithm, an ensemble of decision trees, known for its robustness in handling outliers and noise which is crucial in clinical datasets like ours [19, 25, 26]. The RF approach was preferred over other methods such as GBT due to its inherent resistance to overfitting, particularly important given the high number of predictors in our dataset. Furthermore, the ability of RF to provide a direct measure of variable importance and its internal estimation of generalization error through the out-of-bag error estimate were pivotal reasons for its selection. These features make RF particularly suitable for our study where model interpretability and robustness are essential. The two main parameters in RF, mtry (the number of random variables used in each tree) and ntree (the number of trees used in the forest), were set to the square root of the number of predictors and 500, respectively, to optimize the balance between model accuracy and computational efficiency. The missing values were addressed by median imputation for each variable to minimize bias. The proportions of missing values in variables are presented in Supplementary Material, Table S1.

The dataset was randomly split into two separate data sets using 5-fold cross-validation on the RF method: 80% for the training set to build a fitted model and the remaining 20% for the validation set to obtain unbiased estimates of correct classification rates and variable importance. As the equation is shown below, the correct classification rate (accuracy) was the number of observations that had been correctly classified divided by the sample size.

$$\mathrm{Correct}\;\mathrm{classification}\;\mathrm{rate}\;=\;\frac{\mathrm{True}\;\mathrm{Seizures}\;+\;\mathrm{True}\;\mathrm{IAE}\;+\;\mathrm{True}\;\mathrm{ANE}}{\mathrm{Number}\;\mathrm{of}\;\mathrm{patients}\;\mathrm{in}\;\mathrm{the}\;\mathrm{data}\;\mathrm{set}}$$

### Statistical analysis

Data were expressed as median (interquartile range (IQR)) for non-normally distributed variables and number (percentage) for categorical variables. The normality of the data distribution was examined by using the Shapiro-Wilk tests. Baseline characteristics were compared between patients with seizures/convulsions, IAE and ANE using the Kruskal-Wallis test and Chi-Square/Fisher’s exact test to detect any differences in the continuous and categorical variables. The clinical and laboratory data were compared between the training and validation set using Mann-Whitney U-Test and Chi-Square/Fisher’s exact test. A two-sided *P*-value of < 0.05 was regarded as statistically significant. Data management and statistical analyses were conducted using SAS (version 9.4, SAS Institute Inc.) and R software (version 3.2.5, R Project for Statistical Computing).

## Results

### Characteristics of the patients

**Fig. 1:**
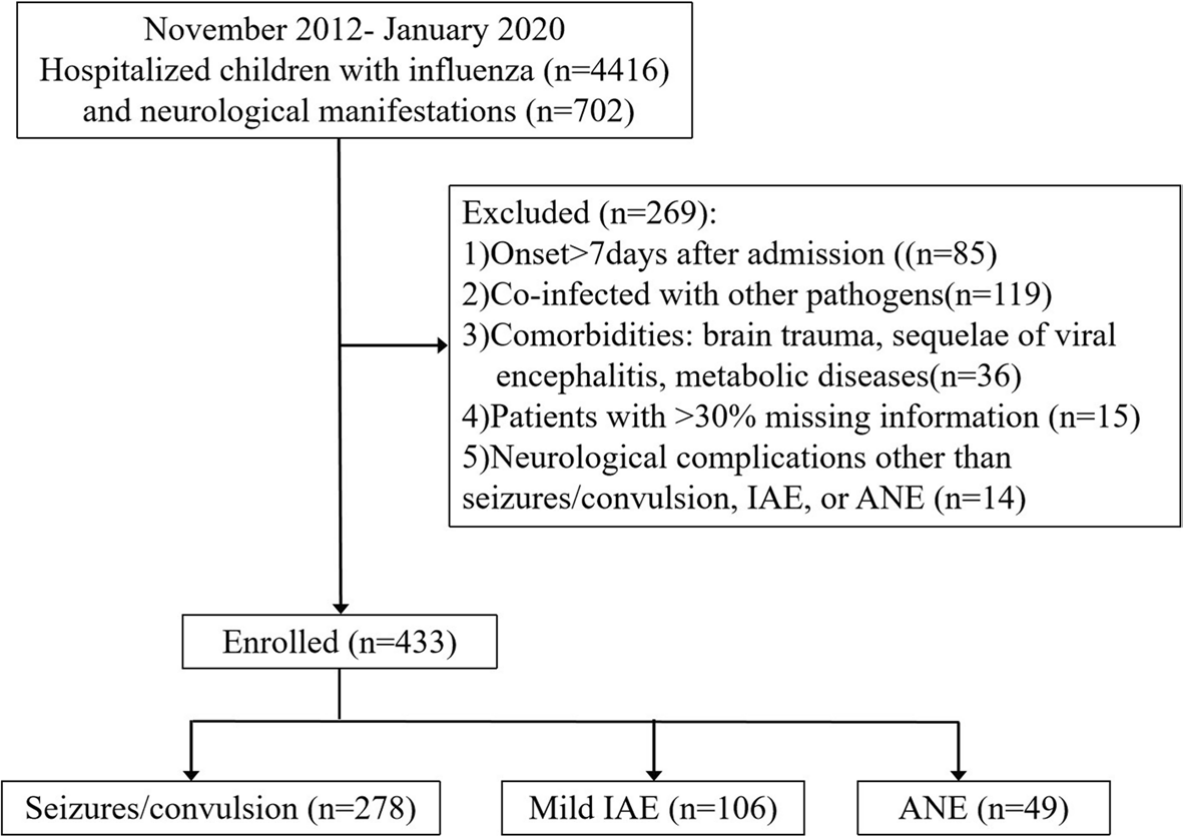
Flowchart of study population. IAE: influenza virus-associated encephalitis; ANE: acute necrotizing encephalopathy

**Fig. 2:**
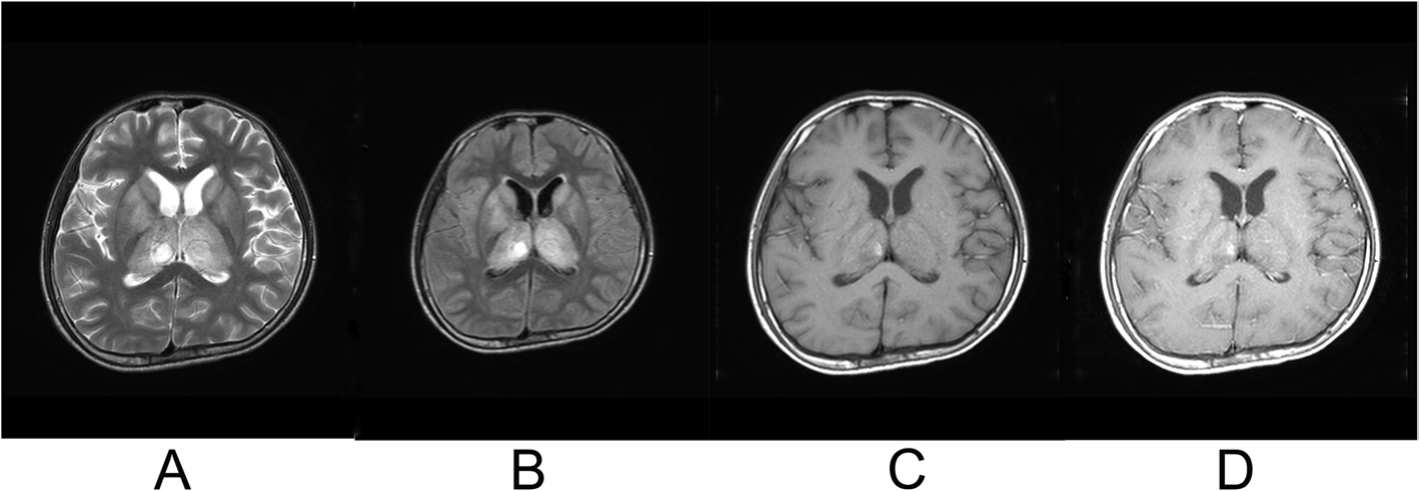
Brain MRI of a 13-year-old boy with acute necrotizing encephalopathy. **A** Axial view of T2-weighted image (T2WI) show swelling and hyperintense signals (arrow) in both thalami; (**B**) Axial view of T2 fluid-attenuated inversion recovery sequence (T2-FLAIR) show swelling and hyperintense signals (arrow) in both thalami; (**C**) equal or slightly low signals on T1-weighted image (T1WI) with internal hemorrhage (arrow) and necrosis (star); (**D**) contrast-enhanced T1WI shows no enhancing brain lesions

A total of 433 patients had met eligibility criteria and enrolled (Fig. 1). The median age of all patients was 2.8 (IQR 1.7–6.1) years, and the majority were male (*n* = 294, 67.89%). Among them, 278 (64.2%) were ultimately diagnosed as seizures/convulsions, 106 (24.5%) as mild IAE, and 49 (11.3%) as ANE. The 78 variables, including demographic characteristics, clinical symptoms, and biochemical and hematologic indicators collected for each patient, are shown in Table 1. The in-hospital mortality rates of the three groups were 0.4% (1/278; this patient had a chromosomal abnormality), 0% (0/106), and 32.7% (16/49) in the seizures/convulsions mild IAE, and ANE groups, respectively. Figure 2 provides a brain MRI of one representative ANE case. All the patients were taken blood samples for examination immediately after admission.

### Variable selection and model development

**Fig. 3:**
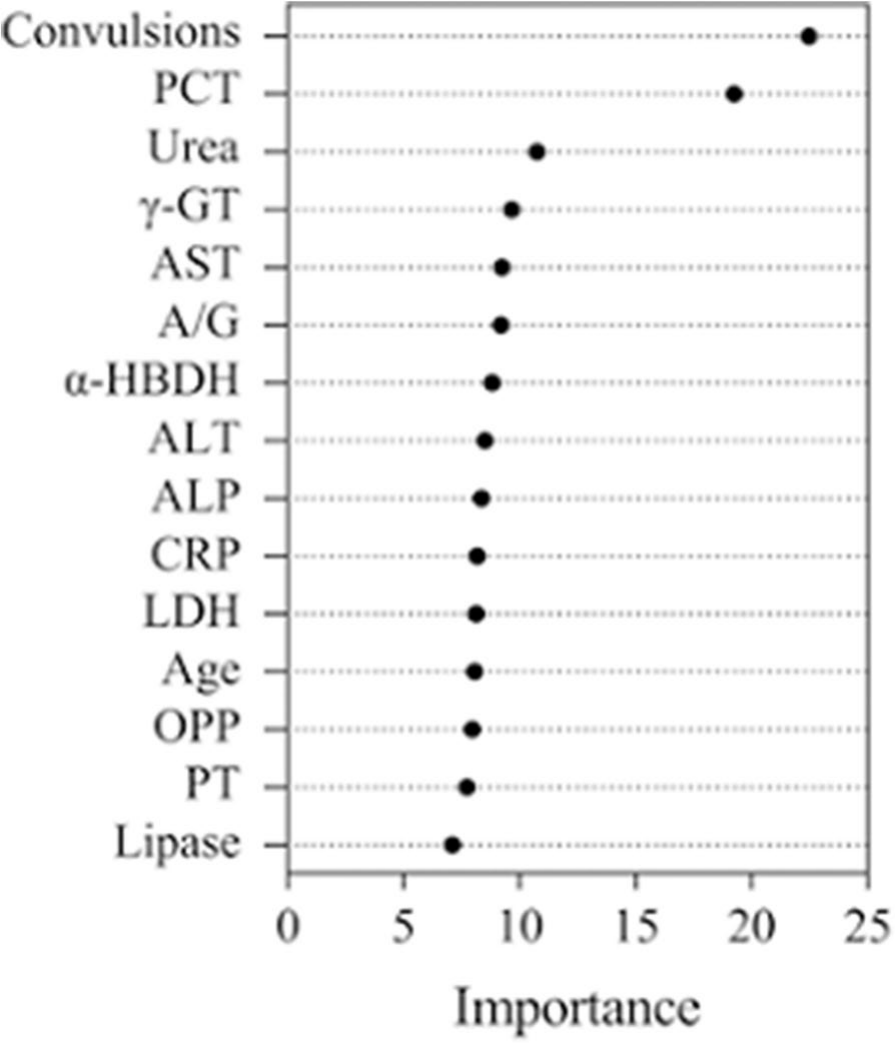
Variable importance of top 15 variables identified by random forest (RF) model. Procalcitonin, PCT; γ-glutamyltransferase, γ-GT; aspartate aminotransferase, AST; α-hydroxybutyric dehydrogenase, α-HBDH; alanine aminotransferase, ALT; alkaline phosphatase, ALP; c-reactive protein, CRP; lactate dehydrogenase, LDH; oxygen partial pressure, OPP; prothrombin time, PT

**Fig. 4:**
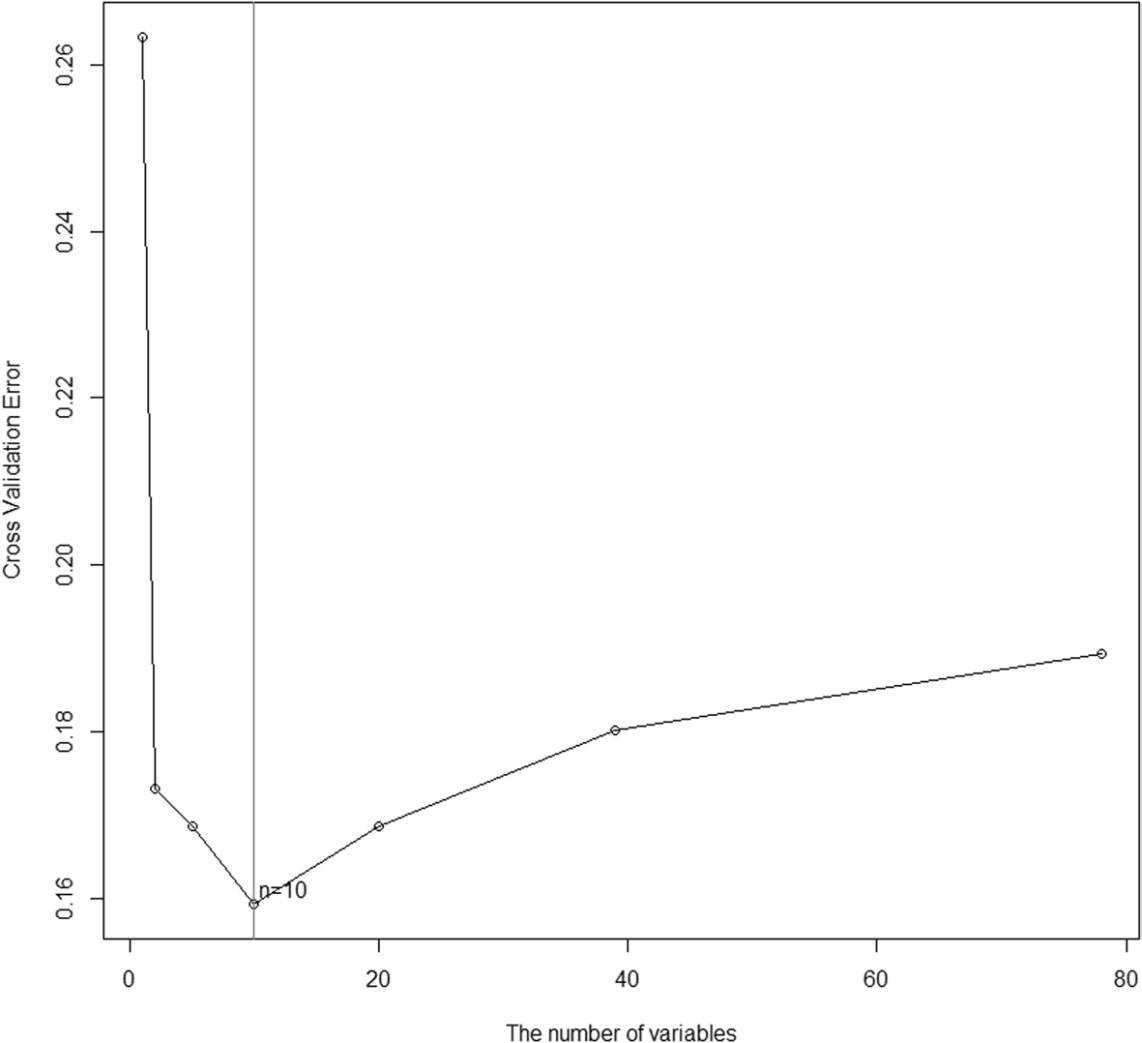
Relationship between the cross-validation error and the number of variables

 Variable selection was carried out using the different subsets of features. The top 15 variables selected in order of their importance are shown in Fig. 3. The higher importance values indicate that the variable has more impact on predictions. Figure 4 shows the relationship between the cross-validation error and the number of variables. The error dropped rapidly at the beginning and then increased gradually with the number of variables. When the number of variables was 10, the minimum error of 0.16 was achieved. Thus, we included 10 features in the model, including convulsions, procalcitonin (PCT), urea, γ-glutamyl transferase (γ-GT), aspartate aminotransferase (AST), albumin/globulin ratio (A/G), α-hydroxybutyric dehydrogenase (α-HBD), alanine aminotransferase (ALT), alkaline phosphatase (ALP), and C-reactive protein (CRP).

### Variable influence

**Fig. 5:**
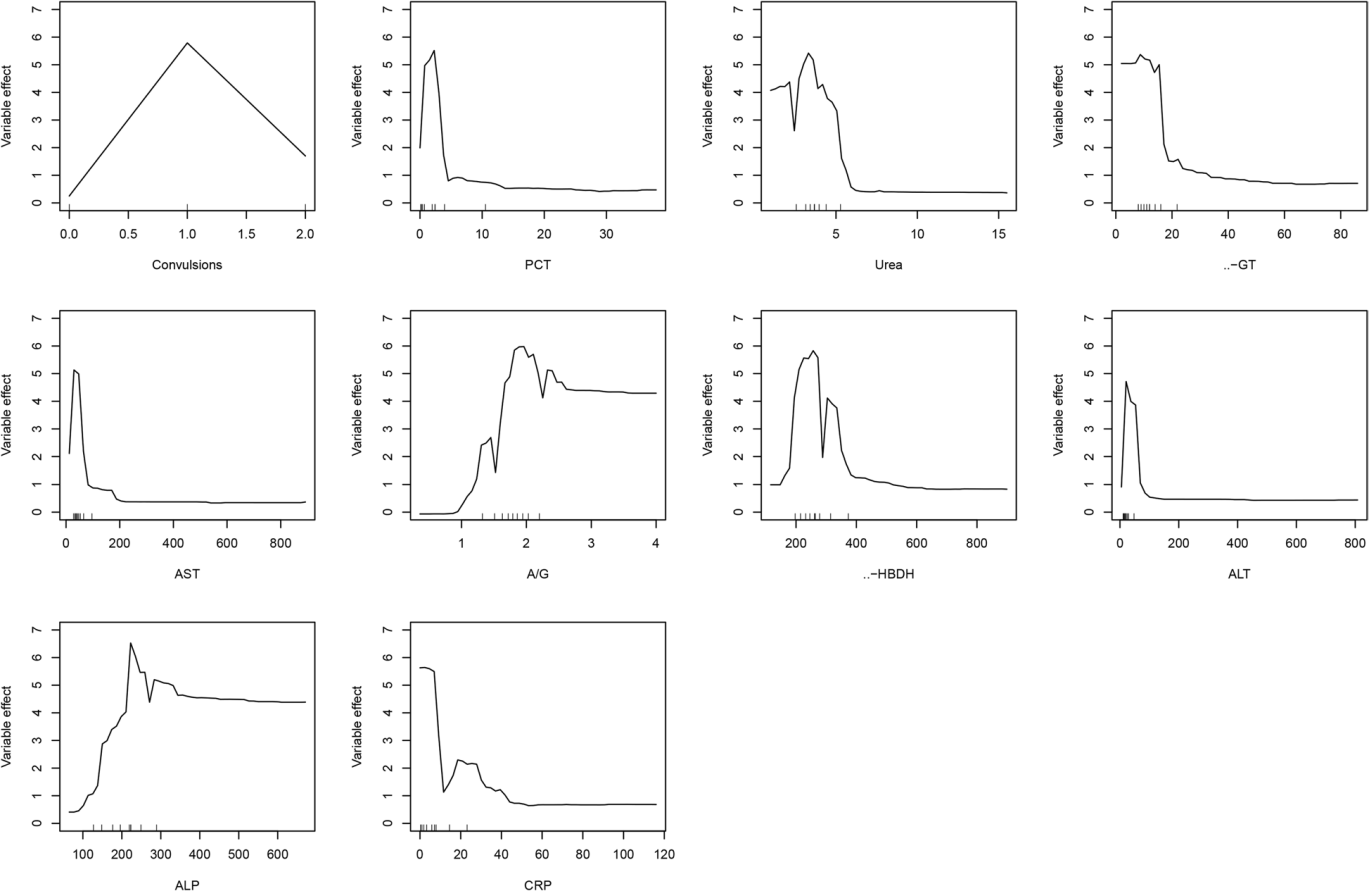
Influence of the selected variables calculated by the random forest method on the seizure. PCT: procalcitonin; γ-GT: γ-glutamyltransferase; AST: aspartate aminotransferase; A/G: albumin/globulin ratio; α-HBDH: α-hydroxybutyric dehydrogenase; ALT: alanine aminotransferase; ALP: alkaline phosphatase; CRP: C-reactive protein

 The independent influences of 10 variables mentioned above on the seizure were calculated by the random forest (Fig. 5). Each variable’s effect, as depicted on the vertical axis, quantifies the change in the outcome predictive accuracy when that specific variable’s value is modified within the model, while keeping other factors constant. The horizontal axis represents the specific levels or values of each variable, which allows us to observe how changes in each variable’s levels are associated with changes in their predictive influence, known as the variable effect. For instance, a low number of convulsions (1–2) at admission generally implies a straightforward diagnosis of seizures without further complications, reflected by a higher variable effect score. Conversely, a higher number of convulsions (≥ 3 or 0) may suggest a more complex clinical scenario such as mild IAE or ANE. Biochemical indicators such as PCT, urea, γ-GT, α-HBDH, ALT, and AST are depicted with their respective effect scores, showcasing their relationship with the likelihood of seizures in the absence of further complications. Similarly, the A/G and ALP levels are illustrated to show their inverse relationship with seizure likelihood.

### Model performance and validation

The prediction model gave a prediction accuracy of 84.2%. In order to obtain unbiased estimates of accuracy, the model was internally validated using 5-fold cross-validation. The 10 variables included in the model and the outcomes were compared between the training and validation sets (Table 2), no significant intergroup differences were observed. When applied to the held-out validation set, the prediction accuracy was 88.2%, indicating the good discriminatory performance of the model.

**Table 2:** Clinical and laboratory data in the training and validation sets

Variable, median (IQR) or as shown	Training set, *n* = 348	Validation set, *n* = 85	*P*
Convulsions, n (%)			0.828
None	41 (11.8)	10 (11.8)	
1 to 2	251 (72.1)	59 (69.4)	
≥ 3	56 (16.1)	16 (18.8)	
PCT	1.9 (0.3,4)	2.2 (0.3,3)	0.993
Urea	3.7 (3.3,4.1)	3.7 (3.4,4.3)	0.249
γ-GT	12 (10,14)	12 (10,15)	0.177
AST	43 (34,59.5)	42 (35,60)	1.000
A/G	1.8 (1.6,2)	1.8 (1.6,2)	0.764
α-HBDH	259 (222,288)	261.1 (236,300)	0.393
ALT	19 (14,26)	19 (14,26)	0.590
ALP	220 (161.5,234)	217 (162,226)	0.768
CRP	5.4 (1.4,9.3)	7.1 (1.4,16.8)	0.149
Neurological complications, n (%)			0.931
Seizure	224 (63.4)	54 (63.5)	
Mild IAE	84 (24.1)	22 (25.9)	
ANE	40 (11.5)	9 (10.6)	

*IQR* Interquartile range, *PCT* Procalcitonin, *γ-GT* γ-glutamyltransferase, *AST* Aspartate aminotransferase, *A/G* Albumin/globulin ratio, α-HBDH α-hydroxybutyric dehydrogenase, *ALT* Alanine aminotransferase, *ALP* Alkaline phosphatase, *CRP* C-reactive protein, *IAE* Influenza virus-associated encephalitis, *ANE* Acute necrotizing encephalopathy

**Fig. 6:**
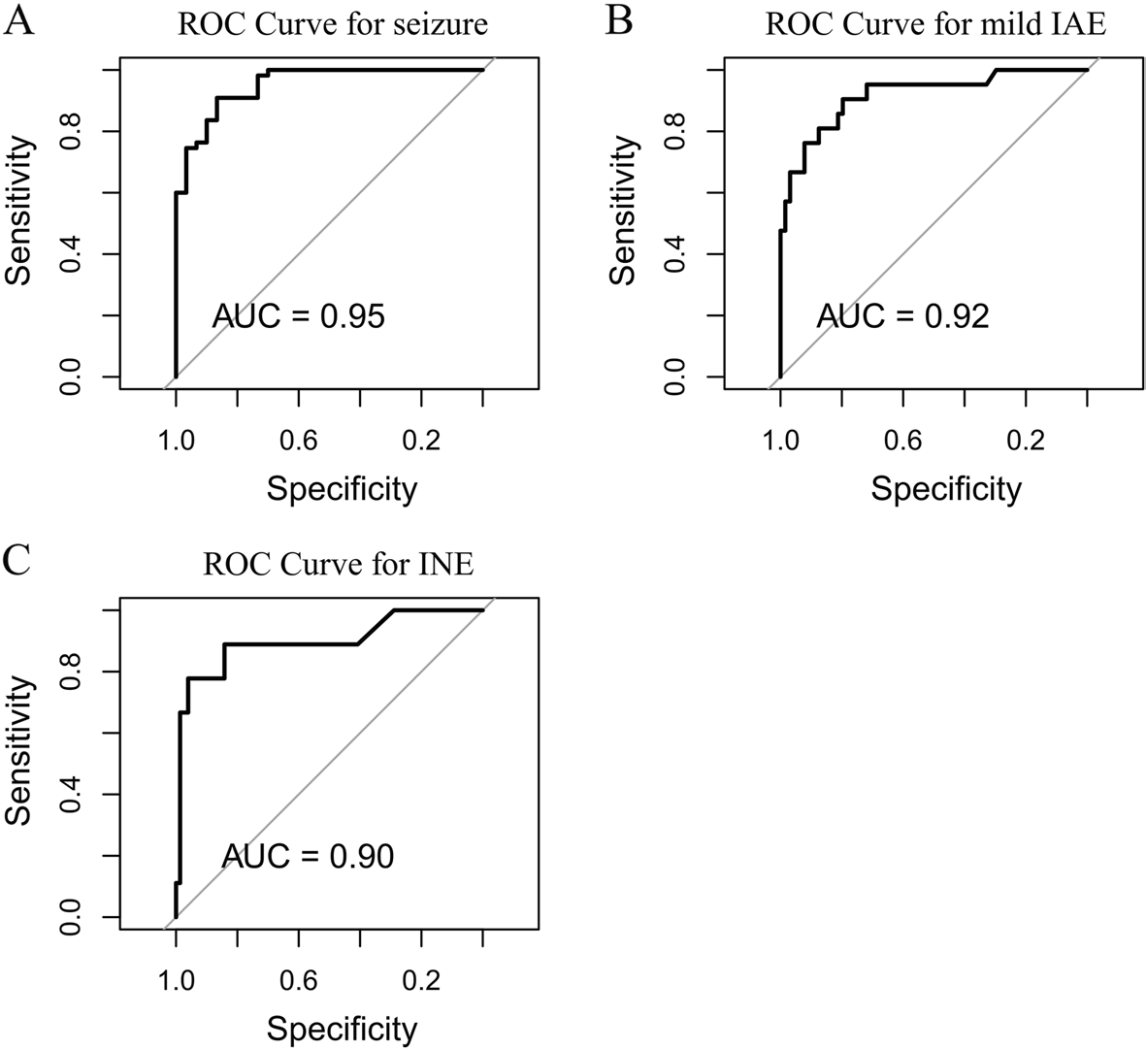
ROC curves on random forest model in validation set. A, ROC curve of classifying seizure from the other two classes. B, ROC curve of classifying mild IAE from the other two classes. C, ROC curve of classifying INE from the other two classes

 In the validation set, seizures/convulsions were less likely to be wrongly classified (3.7%, 2/54), but mild IAE (22.7%, 5/22) was prone to be misdiagnosed as seizures/convulsions, and a small proportion (4.5%, 1/22) of them was prone to be misdiagnosed as ANE (Table 3). Of the children with ANE, 22.2% (2/9) were misdiagnosed as mild IAE, and none were misdiagnosed as seizures. Furthermore, the accuracy of classifying seizure from the other two classes, mild IAE from the other two classes, and INE from the other two classes are 0.95, 0.92, and 0.90, respectively (Fig. 6). This suggests that the model performs better when only used to distinguish between two classes.

**Table 3:** Confusion matrix of the training and validation sets

Actual group	Predicted group				Misclassification
	Seizure	Mild IAE	ANE	Total	
Training set					
Seizure	210	11	3	224	6.3%
Mild IAE	22	57	5	84	32.1%
ANE	7	7	26	40	35.0%
Validation set					
Seizure	52	2	0	54	3.7%
Mild IAE	5	16	1	22	27.3%
ANE	0	2	7	9	22.3%

*IAE* Influenza virus-associated encephalitis, *ANE* Acute necrotizing encephalopathy

## Discussion

Neurological complications caused by influenza are serious conditions, mainly occurring in young children and with high morbidity and mortality rates [27]. Our study developed and internally validated a diagnostic model for distinguishing ANE from seizures/convulsions alone and mild IAE in children with influenza. The first measurements of biochemical and hematologic indicators on admission were evaluated using the RF method, avoiding the problem of model overfitting caused by correlations among the variables. The discrimination performance of the model was satisfactory, with an accuracy above 0.80 from both model development and internal validation. Seizures/convulsions were less likely to be wrongly classified, but mild IAE was prone to be misdiagnosed as seizures/convulsions. Of the children with ANE, around 20% were misdiagnosed as mild IAE, and none were misdiagnosed as seizures/convulsions. Our model, including only 10 common variables, was convenient for clinicians to perform early diagnosis and intervention.

In concordance with the result of previous studies, we found that convulsions, PCT, urea, γ-GT, AST, A/G, α-HBD, ALT, ALP, and CRP were important predictors of ANE. Indeed, ANE is characterized by frequent convulsions [7, 8, 28], as observed in the present study. A previous study showed that a combination of age < 4 years, repeated seizures, altered consciousness, and positive Babinski’s sign were the high-risk factors for ANE [28]. On the other hand, the literature suggests no specific laboratory marker for diagnostic of ANE [29–31], but elevated serum transaminases could be associated with ANE [7, 30]. In the present study, the likelihood of seizures/convulsions outcome decreased with the increasing levels of AST, indicated that the risk of IAE or ANE increased with the increase of AST. Studies showed that AST levels increase when soft-tissue necrosis occurs [32–34]. Early research showed that brain dehydration was maximal 30 min after urea injection and improved cerebral circulation [35]; the increase in urea might be related to the reactive regulation of early cerebral edema. The endothelial cells of the capillaries of the cerebral cortex in rats showed high γ-GT activity [36], suggesting the possibility of cerebrovascular involvement in early ANE. Activities of α-HBDH were measured in rats after intermittent exposure to aerogenic hypoxia but had no effects on adults [37] and were also associated with edema, ischemic and hemorrhagic changes [38]. In the early stages of influenza, increases in these factors could be a high risk for ANE.

In the present study, the imaging parameters were not included in the analyses, mainly because of the broad examination types and protocols. Further studies should consider the possibility of adding imaging variables to refine the present model. Indeed, the presence of brain imaging features is usually associated with a poor prognosis [39–43]. Furthermore, CSF examination in patients with ANE usually reveals increased amounts of proteins [30]. However, the cerebrospinal fluid examination was not performed in all patients. In the present study, older children were more prone to have ANE. The importance of age was ranked 12th, but only the first 10 variables were included in the model based on the cross-validation error minimum principle. The strain or the subtypes of influenza may play an important role in ANE development. However, detection of the strain or the subtypes of influenza may took a long time, which was not routinely tested at our medical center, so it was not included in the forest model. Compared with ANE, the discrimination ability of the model was lower for mild IAE. It could be because mild IAE is an intermediate condition between seizures/convulsions and ANE based on the symptoms [4]. Indeed, IAE is characterized by convulsions and coma [6, 7, 30], and death is not common [4]. Cadaver studies have shown cerebral vascular damage was found in patients with severe IAE, but necrotic changes were not seen [44, 45]. Cerebrovascular involvement could be found in both severe IAE and ANE, that may be the reason of overlap between two of them. In the early stage of disease, the above 10 variables in the forest model can be used to predict severe IAE or ANE, and indicate clinicians to take special examinations evaluating the severity and prognosis of disease, such as thromboelastography [46], indicators of brain tissue necrosis (such as lactate dehydrogenase and malondialdehyde levels in CSF) [47], and a special sequence of brain MRI (e.g., thalamic proton magnetic resonance spectroscopy (MRS) measures [48], DWI and MR angiography). The inspections mentioned above are not routine examinations in the diagnosis of emergency department patients. Futhermore, once the prediction model indicates severe IAE or ANE, clinicians can carry out early intervention such as low brain temperature, antiviral medication, immunoglobulin, glucocorticoids, and plasma exchange, etc [4], as soon as possible to improve the prognosis, and provide objective evidence for communication with the family and obtaining treatment approval.

This study has some limitations. The correct classification rate of seizures is high in our model, but potential misclassification between mild IAE and ANE can not be ignored. The ability of differentiation might be improved by adding imaging characteristics and CSF parameters into the model. Furthermore, as the pathogenesis of IAE and IANE is not fully understood, there is overlap between the two in diagnosis. Therefore, to avoid confusion, this study only recruited the IAE patients without brain imaging abnormalities (defined as the mild IAE group) and the ANE patients (clinical and imaging conforming to the ANE diagnosis), aiming to predict the trend of ANE through the earliest clinical information. In addition, only the first detection values of biochemical and hematological indicators on admission were considered, and the eventual changes in those indicators were not evaluated. Since ANE is a rapidly progressing condition, the exact timing of the evaluations may affect the results. Despite these limitations, we believe the conclusion of the study would not be overturned.

## Conclusion

In the present study, 10 high-risk factors were selected as variables from clinical characteristics and serological indicators, including convulsions, PCT, urea, γ-GT, AST, etc., for developing a prediction model, which could accurately distinguish ANE from seizures/convulsions. This practical diagnostic tool is convenient and provides valuable information for clinicians in choosing early interventions or planning further examination. Nevertheless, the diagnostic accuracy for differentiating IAE from ANE, and differentiating mild IAE from seizures/convulsions, still needs to be improved. Further researches are needed to refine this model.

### Supplementary Information

**Supplementary Material 1.**

## Data Availability

The datasets used and/or analysed during the current study are available from the corresponding author on reasonable request.

## Abbreviations

ANE: Acute necrotizing encephalopathy
IAE: Influenza-associated encephalopathy
CT: Computed tomography
MRI: Magnetic resonance imaging
T1WI: T1-weighted image
DWI: Diffusion-weighted imaging
ADC: Apparent diffusion coefficient
RF: Random forest
CSF: Cerebrospinal fluid
EMRS: Electronic medical records system
IQR: Interquartile interval
PCT: Procalcitonin
γ-GT: γ-glutamyl transferase
AST: Aspartate aminotransferase
A/G: Albumin/globulin ratio
α-HBD: α-hydroxybutyric dehydrogenase
ALT: Alanine aminotransferase
ALP: Alkaline phosphatase
CRP: C-reactive protein
MRS: Magnetic resonance spectroscopy
