# Supplementary material for: Identification of neurological complications in childhood influenza: a random forest model
Source: BMC Pediatr. 2024 May 20;24:347. doi: 10.1186/s12887-024-04773-4 (PMC11103977; doi:10.1186/s12887-024-04773-4)
Supplement: Supplementary file 1 — Supplementary Material 1. [file 12887_2024_4773_MOESM1_ESM.docx]

Additional file 1. The proportion of missing data for the variables.

| Variable | Proportion of missing（%） |
| --- | --- |
| Cholinesterase (U/L) | 25.6 |
| ALT (U/L) | 4.8 |
| γ-GT (U/L) | 5.3 |
| ALP (U/L) | 19.2 |
| Amylase (U/L) | 25.4 |
| AST (U/L) | 5.5 |
| Globulin (g/L) | 5.3 |
| CK-CKMB (U/L) | 19.2 |
| Lactic dehydrogenase (U/L) | 4.8 |
| A/G | 5.3 |
| α-HBDH (U/L) | 9.9 |
| CK (U/L) | 5.3 |
| Calcium (mmol/L) | 5.1 |
| CKMB (U/L) | 5.5 |
| Total protein (g/L) | 5.1 |
| Albumin (g/L) | 5.1 |
| Total bile acid (µmol/L) | 5.5 |
| hs-CRP, (mg/L) | 20.3 |
| Uric acid (µmol/L) | 7.9 |
| IBIL (µmol/L) | 5.3 |
| DBIL (µmol/L) | 5.3 |
| Lipase (U/L) | 25.6 |
| Creatinine (µmol/L) | 5.5 |
| TBIL (µmol/L) | 5.3 |
| Thrombin time (s) | 8.3 |
| Prothrombin time (s) | 8.3 |
| Fibrinogen (g/L) | 8.3 |
| Coagulation activity (%) | 21.7 |
| INR | 8.3 |
| CRP (mg/L) | 17.3 |
| RBC (10^12^/L) | 0.2 |
| Monocytes (10^9^/L) | 0.9 |
| Monocytes% (%) | 0.9 |
| Lymphocytes (10^9^/L) | 0.9 |
| Lymphocytes% (%) | 0.9 |
| Large platelet ratio (%) | 2.8 |
| MCV (fL) | 0.9 |
| Eosinophils (10^9^/L) | 0.9 |
| MPV (fL) | 2.8 |
| RBC distribution width, SD (%) | 0.9 |
| Thrombocytocrit (%) | 2.8 |
| Mean hemoglobin concentration (pg) | 0.9 |
| Neutrophils (10^9^/L) | 0.9 |
| Neutrophils% (%) | 0.9 |
| Blood platelets (10^9^/L) | 0.2 |
| PDW | 2.8 |
| RBC distribution width, CV (%) | 0.9 |
| WBC (10^9^/L) | 0.2 |
| Oxygen partial pressure (kPa) | 4.2 |
| Urea (mmol/L) | 29.3 |
| Procalcitonin (ng/ml) | 28.9 |
| Potassium (mmol/L) | 1.8 |
| BEECF (mmol/L) | 18.0 |
| Carbon dioxide (mmol/L) | 3.2 |
| Hematocrit (%) | 0.0 |
| Standard ionic calcium (mmol/L) | 18.0 |
| Lactic acid (mmol/L) | 4.2 |
| Standard bicarbonate (mmol/L) | 17.8 |
| Whole blood residual base (mmol/L) | 4.4 |
| Calcium ions (mmol/L) | 3.7 |
| Acidity (pH) | 4.6 |
| PCO2 (kPa) | 17.1 |
| Glucose (mmol/L) | 2.5 |
| Sodium (mmol/L) | 1.8 |
| Hemoglobin (g/L) | 0.2 |
| Immunoglobulin A (g/L) | 9.2 |
| Immunoglobulin E (IU/ML) | 24.0 |
| Immunoglobulin G (g/L) | 9.2 |
| Immunoglobulin M (g/L) | 9.2 |
| Complement C3 (g/L) | 22.9 |
| Complement C4 (g/L) | 22.9 |
